# Internet Addiction Phenomenon in Early Adolescents in Hong Kong

**DOI:** 10.1100/2012/104304

**Published:** 2012-06-18

**Authors:** Daniel T. L. Shek, Lu Yu

**Affiliations:** ^1^Department of Applied Social Sciences, The Hong Kong Polytechnic University, Hong Kong; ^2^Public Policy Research Institute, The Hong Kong Polytechnic University, Hong Kong; ^3^Department of Social Work, East China Normal University, Shanghai 200241, China; ^4^Kiang Wu Nursing College of Macau, Macau; ^5^Division of Adolescent Medicine, Department of Pediatrics, Kentucky Children's Hospital, University of Kentucky College of Medicine, Lexington, KY 40506, USA

## Abstract

The present study investigated the prevalence and demographic correlates of Internet addiction in Hong Kong adolescents as well as the change in related behavior at two time points over a one-year interval. Two waves of data were collected from a large sample of students (Wave 1: 3,328 students, age = 12.59 ± 0.74 years; Wave 2: 3,580 students, age = 13.50 ± 0.75 years) at 28 secondary schools in Hong Kong. Comparable to findings at Wave 1 (26.4%), 26.7% of the participants met the criterion of Internet addiction at Wave 2 as measured by Young's 10-item Internet Addiction Test. The behavioral pattern of Internet addiction was basically stable over time. While the predictive effects of demographic variables including age, gender, family economic status, and immigration status were not significant, Internet addictive behaviors at Wave 1 significantly predicted similar behaviors at Wave 2. Students who met the criterion of Internet addiction at Wave 1 were 7.55 times more likely than other students to be classified as Internet addicts at Wave 2. These results suggest that early detection and intervention for Internet addiction should be carried out.

## 1. Introduction

The use of the Internet has brought a variety of convenience to our modern life. Nonetheless, negative impact is also created by addictive behaviors to the Internet pervasively on one's academic and working performance, family life, social relationships, physical health, and psychological well-being [1–3]. Although there are different views on the term, “Internet addiction” or “pathological use of the Internet” usually refers to the phenomenon that an individual is unable to control his or her use of the Internet (including any online-related, compulsive behavior) which eventually causes one's marked distress and functional impairment in daily life [4]. With the soaring number of Internet users, it has been reported that Internet addiction is becoming a serious problem across the world, especially for adolescents. Scholars have also warned that Internet addiction could bring substantial loss of productivity in schools and companies where no Internet governance policies are implemented [5, 6]. As there are few related studies on Internet addiction in Hong Kong, the present study investigated the occurrence and demographic correlates of Internet addiction among a group of Hong Kong adolescents and examined the stability of the phenomenon by comparing the prevalence findings between two time points with a one-year interval.

In the past few decades, several studies have examined the prevalence of youth Internet addiction, with the reported data varying across different areas of the world [7]. It has been found that the occurrence rate of Internet addiction among adolescents ranges from 1.98% to 35.8% in Western and Eastern societies [8–10]. Even in different Chinese communities, prevalence findings of Internet addiction were inconsistent. For example, in Chou and Hsiao's study, 5.9% of Taiwan college students were classified as having Internet addiction [11], whereas Wu and Zhu reported that 10.6% of university students in China mainland could be identified as Internet addicts [12]. While a study on high-school students in Changsha showed a prevalence rate of 2.4% [13], another study in Shanxi revealed that 6.44% of first-year university students were addicted to the Internet [14]. In Hong Kong, using Young's 20-item questionnaire to examine Internet addiction among youth, 61.4% of senior primary school students, 35.2% of Secondary 1 to 3 students, 18.8% of Secondary 4 to 5 students, 35.8% of Secondary 6 to 7 students, and 37.0% of college students were identified as highly at risk of Internet addiction [15]. There were also findings showing that 13.8% of a sample of high school adolescents in Taiwan met the criterion of Internet addiction and had different psychological and psychiatric problems [16].

These inconsistent findings may be explained by several factors on the conceptual and methodological levels. First, various instruments for assessing Internet addiction were used. In Taiwan, researchers tended to use a 40-item Chinese Internet-Related Addictive Behavior Inventory to assess Internet addiction in adolescents [15]. However, Young's questionnaires were usually adopted by scholars in China mainland and Hong Kong [14, 16]. Second, inconsistent diagnostic criteria and cut-off scores were employed in different studies. Although most researchers followed Young's proposed cut-off (i.e., having 4 out of 10 symptoms as the threshold of being classified as Internet addiction), other researchers used a higher cutoff score. Third, some prevalence studies were based on small and unrepresentative samples which limited the generalizability of the findings. Fourth, most of the existing studies utilized cross-sectional designs and thus cannot provide a complete understanding of how Internet addiction developed over time. These problems point to the urgent need to conduct methodologically sound research on youth Internet addiction, particularly in Chinese contexts, where few validated measures exist [17].

Another important puzzle in Internet addiction research is whether an individual's tendency of displaying Internet addictive behaviors remain the same or change over time. On the one hand, some researchers claimed that Internet addiction is a short-term phenomenon which would gradually diminish as time passes [18, 19]. For example, Widyanto and McMurran [18] proposed that Internet addiction is “a temporary phenomenon for some individuals, likely related to the initial novelty of the Internet and wearing off with increased familiarity” (p. 444). Young reported that over half of self-identified “Internet-dependent” had been online for less than one year, suggesting that new users may be more inclined to develop addictive behaviors associated with Internet use [20]. In fact, more than two thirds of “non-Internet-dependent” subjects in Young's study had been online for over a year, which seems to indicate that excessive use of the Internet could be a transient phenomenon that wear off over time in most individuals. There are also perspectives suggesting that real-life difficulties may contribute to Internet addiction because Internet provides an escape for the individual from stressful life events [21]. Once the problems in reality are solved, Internet addictive behaviors would gradually taper off.

On the other hand, another school of thoughts and empirical studies support the stability and persistence of Internet addiction where pathological use of the Internet is believed to be associated with personality factors and other problems. In one study, individuals who were self-reliant, emotionally sensitive, reactive, vigilant, nonconformist, and have low self-disclosure were found to be more likely to become Internet dependent [1]. Amiel and Sargent found that the use of the Internet gave highly neurotic subjects a sense of belonging and made them feel informed, while extraverts tended to use the Internet for instrumental purposes [22]. The comorbidity of Internet addiction and other psychosocial problems provides extra support to expect stability in Internet addiction, although there is no consensus regarding whether Internet addiction should be considered a cause or effect. It was found that lonely individuals used the Internet more frequently and were more likely to use the Internet for emotional support than nonlonely people [23]. All these findings seem to indicate the stability of Internet addiction tendency. However, the studies are severely limited by their cross-sectional design, which collected data only one time and/or examined stability of Internet addiction through retrospective recall technique. Hence, such an approach could only provide a snapshot of Internet addiction. To determine whether Internet addictive behaviors are temporary or stable among adolescents, longitudinal studies examining data across different time points are necessary.

Against the above background, there were two purposes of the present study. The first purpose was to investigate the occurrence rate of Internet addiction and its demographic correlates among a large sample of Hong Kong adolescents using a validated instrument, Young's 10-item Internet Addiction Test [17]. As part of a large longitudinal study on youth development, for which two waves of data have been collected, this paper focuses on data collected at the second wave. Results regarding the first wave of data have been reported elsewhere [24]. To establish causal relationships between different demographic factors and Internet addiction, demographic information (age, gender, family economic status, and immigration) collected at Wave 1 was used to predict youth Internet addictive behaviors at Wave 2. The second purpose was to examine the stability of Internet addiction over time by comparing the occurrence rates of different types of Internet addictive behaviors in the same sample of students at the two time points (Wave 1 and Wave 2). The predictive effect of Internet addiction at Wave 1 on participants' behaviors at Wave 2 was also evaluated after controlling for other demographic variables.

## 2. Methods

The present paper reports findings on participants' Internet addictive behavior collected at the second wave of the longitudinal study. There were 28 secondary schools in Hong Kong participated in this study. Details about the study as well as findings of the first wave of data can be seen in Shek and Yu's paper [24].

### 2.1. Participants

In the school year of 2010-2011, all Secondary 2 students in the selected 28 schools, who participated in the first wave of data collection in the school year of 2009-2010 when they were at Secondary 1 level, were invited to attend the second wave of data collection. There were 3,580 students responding to the questionnaire, including 1,864 males (52.1%) and 1,716 females (47.9%). The mean age of the participants was 13.64 years (SD = 0.75). Local students accounted for 78.6% of the participants; 19.3% of them were born in China Mainland and 2.0% were from other places. The demographic information of the participants is summarized in Table 1. From Wave 1 to Wave 2, data of 2,904 students were successfully matched, indicating an acceptable attrition rate of 12%.

### 2.2. Procedures

The participants were invited to respond to a comprehensive youth development questionnaire including both existing instruments and scales developed by the first author. The questionnaire survey was conducted by a trained research assistant in classroom settings with standardized instructions. At each measurement occasion, the purposes of the study were introduced and confidentiality of the data collected was repeatedly ensured to all participants. School, parental, and student consent had been obtained before data collection. Participants responded to the questionnaires in a self-administered format. The research assistant was present throughout the administration process to answer possible questions from the participants.

### 2.3. Instruments

The questionnaire used in this study comprises questions about participants' Internet addictive behaviors, demographic information, participants' family environment, different measures of youth development constructs, and other problem behaviors. For family factors, participants responded to questions regarding paternal presence, maternal presence, parental marital status, paternal educational level, maternal educational level, and family economic status. Family economic status is indexed by the question of whether the family of the participant is receiving Comprehensive Social Security Assistance (CSSA), a financial aid provided by Hong Kong Government for low-income populations, at the time of survey. The scales used to assess Internet addiction and positive youth development constructs are introduced below.

#### 2.3.1. Young's 10-Item Internet Addiction Test (IAT)

Young developed several instruments to assess Internet addiction, among which the 10-item Internet Addiction Test was validated by Shek et al. [17] for Chinese populations and was selected to measure youth Internet addictive behavior in this study. The 10-item IAT asks respondent to answer “Yes” or “No” as to whether they have the listed Internet addictive behaviors in the past one year. Example items include “feeling a need to spend more and more time online to achieve satisfaction” and “feeling restless or irritable when attempting to cut down or stop online use.” A person is classified as “Internet addiction” if he/she shows 4 or more of the listed behaviors. Cronbach's alpha of IAT for the present sample was 0.79 and 0.80 at Wave 1 and Wave 2, respectively.

#### 2.3.2. Chinese Positive Youth Development Scale (CPYDS)

The CPYDS consists of 15 subscales which are listed as follows:

1. Bonding subscale (three items).
2. Resilience subscale (three items).
3. Social competence subscale (three items).
4. Emotional competence subscale (three items).
5. Cognitive competence subscale (three items).
6. Behavioral competence subscale (three items).
7. Moral competence subscale (three items).
8. Self-determination subscale (three items).
9. Self-efficacy subscale (two items).
10. Beliefs in the future subscale (three items).
11. Clear and positive identity subscale (three items).
12. Spirituality subscale (three items).
13. Prosocial involvement subscale (three items).
14. Prosocial norms subscale (three items).
15. Recognition for positive pehavior subscale (three items).

Although the administered questionnaire includes the CPYDQ, findings regarding this scale will be reported elsewhere. The present paper only focused on the descriptive profile of Internet addictive behavior and its demographic correlates as well as the change of behavior over time.

### 2.4. Data Analytic Plan

First, to examine the prevalence of Internet addiction among Hong Kong adolescents, numbers and percentages of adolescents who reported different addictive behaviors associated with Internet use at Wave 2 were computed. Secondly, to investigate the stability or change in participants' Internet addiction over a one-year interval, the percentages of participants showing Internet addictive behaviors at Wave 2 were compared with the percentages found at Wave 1 by using related samples McNemar Tests, a statistic method examining the difference between paired proportions. Participants' IAT scale score at the two waves were also compared with a paired-samples *t*-test.

Thirdly, to investigate the predictive effects of different demographic variables and Internet addictive behaviors at Wave 1 on participants' Internet addiction at Wave 2, both multiple regression analysis and logistic regression analysis were performed. In particular, in the multiple regression analysis, participants' scale score on IAT at Wave 2 served as the dependent variable. For independent variables, gender, and age, were entered in the first block; family economic status and immigration status were entered in the second block; and participants' score on IAT at Wave 1 was input in the third block. For the logistic regression model, whether the participant met the criterion of Internet addiction at Wave 2 was entered as dependent variable; independent variables and their order of input were the same as those in the linear regression model, except that in the third block participants' IAT score was replaced by their eligibility of being classified as Internet addiction at Wave 1 as the predictor.

As immigrant youth from other places than mainland China only accounted for 2.0% of the participants, they were not included in the regression analyses. In other words, the present study only focused on comparing local and immigrant adolescents from mainland China on their Internet addictive behaviors.

## 3. Results

### 3.1. Descriptive Profiles on Internet Addictive Behavior

Numbers and percentages of participants who displayed Internet addictive behaviors in the past one year are summarized in Table 2. Several observations can be highlighted from the findings. First, signs of Internet addiction were still common among Secondary 2 students in Hong Kong. There were 41.1% of the respondents reporting “feeling preoccupied with the Internet or online services and think about it while offline”; 46.6% of the students “stay online longer than originally intended”; 31.6% of the participants “feeling a need to spend more and more time online to achieve satisfaction.” Second, according to Young's criterion, 26.7% of the respondents could be classified as Internet addicted. Third, psychosocial problems related to excessive Internet use were observed: 20.2% of the participants reported to “go online to escape problems or relieve feelings such as helplessness, guilt, anxiety or depression”; 19.4% of the students were found to “lie to family members or friends to conceal excessive Internet use.” These observations suggest that Internet addiction is a serious and widespread problem in Secondary 2 students in Hong Kong which requires more public attention from the Hong Kong society.

### 3.2. Comparison of Internet Addiction over One Year

The differences in percentages of participants with different Internet addictive behaviors on IAT were examined between the two waves of data collection. As can be seen in Table 2, the occurrence rates for eight out of ten Internet addictive behaviors were similar among students at Wave 1 and Wave 2. No significant differences were found in the percentages of students displaying these behaviors. The proportion of students who met the criterion of Internet addiction at Wave 2 (26.7%) was also comparable to that of last year (26.4%). In addition, the result of paired-samples *t*-test showed there was no significant difference (*P* > .05) in students' mean scores on IAT at Wave 1 (Mean = 1.23) and Wave 2 (Mean = 1.24). Overall, these figures suggest that adolescent Internet addiction is a relatively stable phenomenon as opposed to wearing off over time.

It should be noted that, for two individual items, significant differences were detected, with a larger proportion of students at Wave 2 reported that they “stay online longer than originally intended” (*P* < .001) and “risk the loss of a significant relationship, job, or educational or career opportunity because of online use” (*P* = .02) than at Wave 1. Such an increasing tendency may serve as a warning for researchers and practitioners in the field that, without effective intervention/prevention strategies, it is possible that Internet addiction in adolescents would deteriorate with the increasingly wide application of the Internet in youth life.

### 3.3. Prediction of Internet Addictive Behavior

The predictive effects of participants' demographic variables and prior Internet addictive behaviors on pathological use of Internet at Wave 2 were examined. Tables 3 and 4 present the results of multiple regression analysis and logistic regression analysis, respectively.

Several findings can be observed from the results of multiple regression analysis. First, participants' age failed to predict Internet abuse behaviors. Second, gender was not related to adolescent Internet addiction: boys and girls had similar IAT scale scores. Third, both family economic status and immigration status had no significant influence on participants' addictive behaviors associated with Internet use. Fourth, after controlling for the demographic variables, participants' pathological use of Internet at Wave 1 significantly predicted Internet addictive behavior at Wave 2. The whole model explained 31% of the variance in adolescents' IAT scores at Wave 2, which was almost all from participants' prior Internet addictive behaviors.

Using the probability of being classified as Internet addiction as the dependent variable, results of logistic regression analysis again showed that previous Internet addiction was the only significant predictor of whether a participant would meet Young's criterion of Internet addiction at Wave 2 (*B* = 2.02, odds ratio = 7.55, *P* < .001). In other words, students who met the criterion of Internet addiction at Wave 1 were 7.55 times more likely to be identified as having Internet addiction at Wave 2. The nonsignificant findings regarding demographic predictors indicated that for the present sample of students, the occurrence of Internet addiction was unrelated to their age, gender, family economic status, and immigration status. These findings are consistent with the results of linear regression analysis.

## 4. Discussion

This study investigated the prevalence and demographic correlates of Internet addictive behaviors in Hong Kong adolescents and examined stability and change in pathological use of Internet as measured by Young's 10-item Internet Addiction Test. A sample of more than 3,000 secondary school students was assessed twice over one year. The results showed that Internet addiction appeared to be a common problem in Hong Kong adolescents which may need more public attention and resources to develop effective prevention/intervention strategies. The two-wave longitudinal findings lent support for stability in Internet addiction as opposed to wearing off over time. Specifically, the percentages of participants who met the criterion of Internet addiction were comparable across one year. While none of the demographic variables in the present study predicted Internet addiction, one's earlier pathological use of the Internet significantly affected the individual's later Internet addictive behaviors and the probability of being classified as Internet addicts. Clearly, these findings suggest the importance of early detection and intervention for Internet addiction in Chinese adolescents in Hong Kong.

Consistent with the findings at Wave 1 [24], more than one fourth of the participants (26.7%) at Wave 2 were identified as having Internet addiction based on Young's criterion. The percentages of adolescents showing various Internet misuse behaviors ranged from 11.0% to 46.6%. When the two-wave comparison was made, no significant differences were detected for most addictive behaviors and for participants' IAT scale scores. These findings support the stability in Internet addiction. In other words, adolescents' Internet addictive behavior is not a transient phenomenon that will naturally disappear as adolescents grow older. As such, effective strategies must be developed and implemented to help youth control excessive use of the Internet and form healthy habit associated with Internet use. At the same time, it should be noted that the occurrence rate of Internet addiction found in this study is higher than previously reported prevalence data on Hong Kong adolescents by other researchers [17, 25]. Also, there were more students at Wave 2 reported that they “stay online longer than originally intended” (46.6%) and “risk the loss of a significant relationship, job, or educational or career opportunity because of online use” (22.3%), as compared to Wave 1. This appears to be a worrying tendency. As the Internet has increasingly become an important part of adolescent life, the risk of youth being addicted to the Internet also increases. In the present study, the findings that more adolescents reported excessive time spent on Internet activities and showed impaired social relationships caused by Internet use after one year actually suggest a growing severity of the issue. To solve the problem, researchers, educators, parents, and policy makers must work together and act promptly. For example, school-based prevention programs that involve the participation of parents, teachers, and students should be implemented. The government could promote public awareness of the seriousness of Internet addiction and its effects on society through major media and provide support for different agencies to conduct scientific research and develop effective strategies to prevent Internet addiction.

There are both good and bad implications regarding the relatively stable Internet addiction picture in Secondary 1 and Secondary 2 students in Hong Kong. With specific reference to the “good” implication, stability in the level as well as the prevalence of Internet addiction suggests that there is no further deterioration of the situation, which is quite unlike other adolescent risk behaviors, such as substance abuse, delinquency, and intention to engage in problem behavior in the future. Theoretically, there is a need to understand why Internet addiction does not deteriorate in adolescents over time. Is it due to the fact that the baseline prevalence is already very high, as compared to other youth risk behaviors, or that adolescents would start to have more self-control as they become more mature? Concerning the “bad” implication, adolescents' maintenance of relatively same rate of Internet addiction over time suggests that developmental maturation alone may not be able to reduce Internet addictive behavior. Apparently, if we want to reduce the severity of the problem, there is a need to provide additional intervention strategies.

Interestingly, all demographic variables, including gender, age, immigration status, and family economic status, failed to predict youth Internet addiction in this study. This means that students with different demographic background are at similar risk of developing Internet addictive behaviors. Except for the prediction of age on Internet addiction, the present finding further confirmed the results obtained from the same sample of students one year ago [24]. The study on Wave 1 data showed that older students displayed more pathological use of the Internet, which can be explained by older students' greater developmental dynamics, such as a stronger need to develop a sense of identity, and more access to the Internet, than younger students when they first entered into secondary schools. It may be that after one-year secondary school life, such discrepancies between older and younger students are reduced. Besides, participants in the present study were all at the same grade. The variance in student age was relatively small, which may also contribute to the nonsignificant effect of age on Internet addictive behaviors. Future studies should recruit participants of a wider range of age to further establish the relationship between age and Internet addiction. In addition, in contrast to other addictive behaviors where males usually show a higher level of addictive behavior than do females, the occurrence of Internet addictive behavior appeared to be similar for both adolescent boys and girls. It is suggested that future studies should be conducted to examine gender differences over a longer period of time. In fact, in view of the lack of significant findings for all sociodemographic correlates, it would be important to examine whether this observation could be replicated across time and populations.

Adolescents' prior addictive behaviors associated with the Internet (Wave 1) significantly predicted their Internet addiction after one year, with all possible influence of demographic factors being excluded. The effect size was quite large. Students who met the criterion of Internet addiction at Wave 1 were 7.55 times more likely to be classified as Internet addict one year later. This further suggests that Internet addiction is a relatively stable behavioral pattern. As with other addictive behaviors, once the pattern of pathological use of the Internet is established, it may not be easily changed. The implication is that Internet addiction must be treated and prevented as early as possible. Researchers have proposed different intervention strategies to treat Internet addiction, such as cognitive behavior therapy and motivational enhancement therapy [26]. For example, in cognitive behavior therapy, addicts are taught to identify the distorted thoughts that trigger Internet addictive behaviors and are provided coping strategies trainings to help them effectively deal with real or perceived problems. Motivational enhancement therapy allows the Internet abusers and therapists to collaborate on treatment plans and set achievable goals [26]. Young [4] also suggested several specific treatment techniques, like construct new schedule for using the Internet, set clear and achievable goals to give the addict a sense of control and, provide social support to decrease addicts' dependence on the Internet and family therapy.

While these techniques have been demonstrated to be effective in treating Internet addicts, what is more important is to prevent adolescents who have not met the criterion of Internet addiction to develop such a behavioral pattern. Based on the problem behavior theory that Internet addiction is an intersection of multiple physical, psychological, and technological phenomena instead of a single problem, it has been suggested that prevention programs directed at the organization of different problem behaviors (e.g., substance use, delinquency) may be more appropriate than those target at specific behaviors alone [3], such as promoting positive youth development among adolescents. In fact, based on a longitudinal randomized controlled group trial, researchers have reported that participants of the Project P.A.T.H.S., a program that aims to promote holistic positive development among adolescents, displayed stronger ability to control Internet use than did the comparison group [27]. It seems that positive youth development program represents a promising direction for youth Internet addiction prevention in the future [28, 29]. In the recent longitudinal study including eight waves of data collected in the Project P.A.T.H.S. (a positive youth development program in Hong Kong), results showed that relative to the control group participants, students in the experimental schools (i.e., students participated in the Project P.A.T.H.S.) showed higher levels of psychosocial competencies and less problem behaviors. It is argued that promotion of psychosocial competencies may help to protect young people from risk behavior by enhancing their inner strengths [30–32]. Besides, as different youth risk behaviors tend to coexist, reduction of other problem behaviors, such as intention to engage in risk behavior, may also lower the risk of developing Internet addiction in the long run [33, 34].

Several limitations of this study should be noted. First, Internet addictive behaviors were assessed at only two time points over a relatively short period. Long-term developmental tendency of the behavior cannot be determined. Second, participant age varied within a small range, which limits the investigation of the relationship between age and Internet addiction. Third, single items were used to assess demographic variables, such as family economic status and immigration status. The demographic background of the students may not be fully reflected by these indicators. Ideally, future studies should collect multiple waves of data from adolescents at different age groups, such as students at different secondary school grades, and use more specific demographic index, like monthly family income or years of living in Hong Kong for immigrant students. Fourth, only ten items with dichotomous responses were used to assess Internet addiction, which may produce a less discriminating response profile. More comprehensive measures on Internet addiction may be employed in further research. Despite the limitations, the present findings provide a useful addition to existing literature of Internet addiction in Hong Kong adolescents.

## Figures and Tables

**Table 1:** Descriptive statistics about participants.

Categorical variables	*n*	%		
Gender				
Male	1,864	52.1%		
Female	1,716	47.9%		
Place of birth				
Hong Kong	2,806	78.6%		
Mainland China	690	19.3%		
Others	73	2.0%		
Parental marital status				
First marriage	2,985	82.7%		
Divorced	256	7.1%		
Separated	78	2.2%		
Remarried	168	4.7%		
Others (not first marriage)	122	3.4%		
Family economic status				
Receiving CSSA	208	5.8%		
Not receiving CSSA	2,932	81.2%		
Others (don't know)	472	13.1%		

Continuous variables	Mean	SD	Range	Cronbach's *α*

Age	13.64	0.75	10–17	—
NET-Wave 1	1.23	0.24	1-2	0.79
NET-Wave 2	1.24	0.25	1-2	0.80

Notes. CSSA: Comprehensive Social Security Assistance.

NET-Wave 1: Internet Addiction Test scale score at wave 1.

NET-Wave 2: Internet Addiction Test scale score at wave 2.

**Table 2:** Percentage of participants with Internet addiction behavior in two years.

Internet use behaviors in the past year	No (Wave 2)		Yes (Wave 2)		Yes (Wave 1)		Related-Samples McNemar Tests	
	Number	Percent	Number	Percent	Number	Percent	Statistics	*P*
(1) Feeling preoccupied with the Internet or online services and think about it while offline	2141	58.9%	1494	41.1%	1324	39.9%	1.50	.22
(2) Feeling a need to spend more and more time online to achieve satisfaction	2484	68.4%	1147	31.6%	1072	32.3%	1.11	.29
(3) Unable to control your online use	2765	68.1%	866	23.9%	752	22.7%	0.54	.46
(4) Feeling restless or irritable when attempting to cut down or stop online use	3119	85.9%	511	14.1%	484	14.6%	0.04	.85
**(5) Stay online longer than originally intended**	**1937**	**53.4%**	**1691**	**46.6%**	**1404**	**42.4%**	**14.82**	**.00**
**(6) Risk the loss of a significant relationship, job, or educational or career opportunity because of online use**	**2821**	**77.7%**	**809**	**22.3%**	**644**	**19.5%**	**5.89**	**.02**
(7) Lie to family members or friends to conceal excessive Internet use	2925	80.6%	703	19.4%	651	19.7%	0.51	.48
(8) Go online to escape problems or relieve feelings such as helplessness, guilt, anxiety, or depression	2892	79.8%	732	20.2%	633	19.2%	1.63	.20
(9) Showing withdrawal when offline, such as increased depression, moodiness, or irritability	3151	77.6%	477	13.1%	395	12.0%	1.40	.24
(10) Keep on using Internet even after spending too much money on online fees	3219	89.0%	399	11.0%	331	10.1%	0.10	.75

Participants can be classified as Internet addiction (Young's criteria)	2663	73.3%	972	26.7%	869	26.4%	0.10	.76

Note. Related-Samples McNemar Tests were conducted to examine whether the difference between the distribution of students with Internet addictive behaviors in Wave 1 and Wave 2 is significant.

**Table 3:** Multiple regression analyses on students' Internet use behavior.

	*B*	Beta	Sig	*R* ^2^	*R* ^2^ change
First block					
Age	−0.01	−0.03	0.11		
Gender	0.00	0.00	0.89	0.00	0.00
Second block					
Immigration status	−0.01	−0.01	0.51		
Family economic status	−0.01	−0.02	0.31	0.00	0.00
Third block					
**NET-Wave 1**	**0.57**	**0.56**	**0.00**	**0.31**	0.31**

Notes. *P < .01,  **P < .001.

Dependent variable: NET-Wave 2: Internet Addiction Test scale score at wave 2.

Gender: 1= female; 0 = male.

Immigration status: 1 = immigrant student; 0 = local student.

Family economic status: 1 = Receiving Comprehensive Social Security Assistance (CSSA); 2 = not receiving CSSA.

NET-Wave 1: Internet Addiction Test scale score at wave 1.

**Table 4:** Logistic regression analyses on students' Internet addiction.

	*B*	Odds ratio	*P*
First block			
Age	−0.04	0.96	.59
Gender	−0.13	0.88	.18
Second block			
Immigration status	0.19	1.21	.17
Family economic status (CSSA)	0.09	1.10	.66
Third block			
** IA-Wave 1**	**2.02**	**7.55**	**.00**

Notes. Dependent variable: IA-Wave 2: whether the student meets the criterion of Internet addiction at wave 2.

Gender: 1 = female; 0 = male.

Immigration status: 1 = immigrant students; 0 = local student.

Family economic status: 1 = Receiving Comprehensive Social Security Assistance (CSSA); 2 = not receiving CSSA.

IA-Wave 1: 1= the student met the criterion of Internet addiction at wave 1; 0 = the student did not meet the criterion of Internet addiction at wave 1.
